# Treatment With an Angiopoietin-1 Mimetic Peptide Improves Cognitive Outcome in Rats With Vascular Dementia

**DOI:** 10.3389/fncel.2022.869710

**Published:** 2022-05-06

**Authors:** Lauren Culmone, Brianna Powell, Julie Landschoot-Ward, Alex Zacharek, Huanjia Gao, Elizabeth L. Findeis, Ayesha Malik, Mei Lu, Michael Chopp, Poornima Venkat

**Affiliations:** ^1^Department of Neurology, Henry Ford Hospital, Detroit, MI, United States; ^2^Public Health Sciences, Henry Ford Hospital, Detroit, MI, United States; ^3^Department of Physics, Oakland University, Rochester, MI, United States

**Keywords:** angiopoietin-1, cognition, microinfarct dementia, vasculotide, vascular dementia (VaD), white matter remodeling

## Abstract

**Background and Purpose:**

Vascular dementia (VaD) is a complex neurodegenerative disease affecting cognition and memory. There is a lack of approved pharmacological treatments specifically for VaD. In this study, we investigate the therapeutic effects of AV-001, a Tie2 receptor agonist, in middle-aged rats subjected to a multiple microinfarct (MMI) model of VaD.

**Methods:**

Male, 10–12 month-old, Wistar rats were employed. The following experimental groups were used: Sham, MMI, MMI+1 μg/Kg AV-001, MMI+3 μg/Kg AV-001, MMI+6 μg/Kg AV-001. AV-001 treatment was initiated at 1 day after MMI and administered once daily *via* intraperitoneal injection. An investigator blinded to the experimental groups conducted a battery of neuro-cognitive tests including modified neurological severity score (mNSS) test, novel object recognition test, novel odor recognition test, three chamber social interaction test, and Morris water maze test. Rats were sacrificed at 6 weeks after MMI.

**Results:**

There was no mortality observed after 1, 3, or 6 μg/Kg AV-001 treatment in middle-aged rats subjected to MMI. AV-001 treatment (1, 3, or 6 μg/Kg) does not significantly alter blood pressure or heart rate at 6 weeks after MMI compared to baseline values or the MMI control group. Treatment of MMI with 1 or 3 μg/Kg AV-001 treatment does not significantly alter body weight compared to Sham or MMI control group. While 6 μg/Kg AV-001 treated group exhibit significantly lower body weight compared to Sham and MMI control group, the weight loss is evident starting at 1 day after MMI when treatment was initiated and is not significantly different compared to its baseline values at day 0 or day 1 after MMI. AV-001 treatment significantly decreases serum alanine aminotransferase, serum creatinine, and serum troponin I levels compared to the MMI control group; however, all values are within normal range. MMI induces mild neurological deficits in middle-aged rats indicated by low mNSS scores (<6 on a scale of 0–18). Compared to control MMI group, 1 μg/Kg AV-001 treatment group did not exhibit significantly different mNSS scores, while 3 and 6 μg/Kg AV-001 treatment induced significantly worse mNSS scores on days 21–42 and 14–42 after MMI, respectively. MMI in middle-aged rats induces significant cognitive impairment including short-term memory loss, long-term memory loss, reduced preference for social novelty and impaired spatial learning and memory compared to sham control rats. Rats treated with 1 μg/Kg AV-001 exhibit significantly improved short-term and long-term memory, increased preference for social novelty, and improved spatial learning and memory compared to MMI rats. Treatment with 3 μg/Kg AV-001 improves short-term memory and preference for social novelty but does not improve long-term memory or spatial learning and memory compared to MMI rats. Treatment with 6 μg/Kg AV-001 improves only long-term memory compared to MMI rats. Thus, 1 μg/Kg AV-001 treatment was selected as an optimal dose. Treatment of middle-aged rats subjected to MMI with 1 μg/Kg AV-001 significantly increases axon density, myelin density and myelin thickness in the corpus callosum, as well as increases synaptic protein expression, neuronal branching and dendritic spine density in the cortex, oligodendrocytes and oligodendrocyte progenitor cell number in the cortex and striatum and promotes neurogenesis in the subventricular zone compared to control MMI rats.

**Conclusions:**

In this study, we present AV-001 as a novel therapeutic agent to improve cognitive function and reduce white matter injury in middle aged-rats subjected to a MMI model of VaD. Treatment of MMI with 1 μg/Kg AV-001 significantly improves cognitive function, and increases axon density, remyelination and neuroplasticity in the brain of middle-aged rats.

## Introduction

Dementia is characterized by a progressive deterioration of cognition and memory. Impaired ability to communicate, think and make decisions in addition to memory loss often limits a patient’s capacity to live independently thereby, increasing caregiver responsibilities and socio-economic costs. While Alzheimer’s disease (AD) is the leading cause of dementia, vascular dementia (VaD) due to cerebrovascular disease alone or in combination with AD as mixed dementia remains the next common cause of dementia (Zhao et al., 2020). Reduced blood flow due to a stroke, a series of mini strokes, or microinfarcts in the brain contributes to progressive cognitive deficits in VaD. The risk of developing VaD grows exponentially after the age of 65 particularly in individuals with high risk of cardiovascular disease due to smoking or chronic conditions such as diabetes and hypertension (Gorelick et al., 2011; Zhao et al., 2020). As the global aging population continues to grow, development of therapeutics for VaD becomes increasingly necessary.

Spontaneous cerebral emboli of both cardiac and vascular origin are common among VaD and AD patients and are associated with the occurrence of microinfarcts leading to the rapid progression of dementia and deterioration of cognitive function (Goldberg et al., 2012; Purandare et al., 2012). While microinfarcts comprise a small fragment of total brain volume, the volume of cortical tissue with functional deficits may be as much as 12 times the microinfarct core volume due to impaired hemodynamic responses in peri-infarct tissue, neurovascular uncoupling and disruption of neuronal circuitry (Summers et al., 2017). The brain has substantial tolerance to microemboli, however, upon repeated embolization or depending on size of emboli, distal penetrating arteries are obstructed inducing microinfarcts while a significant proportion of emboli remain in the larger pial vasculature where collateral blood flow may compensate for blocked microvessels (Zhu et al., 2012). Multiple microinfarctions (MMI) can be induced in animals using various methods including the injection of cholesterol crystals, thromboemboli, and microspheres (Venkat et al., 2015). In the current study, we employ cholesterol crystal embolization to induce VaD. This model mimics the rupture of atherosclerotic plaque in patients thereby creating a shower of cholesterol crystals which lodge into the carotid artery and block deep penetrating arterioles causing cerebral microinfarcts. Various studies have demonstrated that unilateral carotid artery injection of cholesterol crystals induces diffuse microinfarcts in the cortex, subcortical tissue, and hippocampus and these microinfarcts were associated with focal blood brain barrier (BBB) disruption and reactive gliosis in the infarct core and surrounding parenchyma (Rapp et al., 2008a, b; Wang et al., 2012, 2017; Venkat et al., 2017, 2019; Yu et al., 2019). White matter (WM) damage and delayed and progressive neuronal loss were associated with cognitive deficits observed as early as day 7 and at least until 4 weeks after microinfarction (Wang et al., 2012; Venkat et al., 2017; Yu et al., 2019).

AV-001 is structurally, functionally, and pharmacologically similar to the predecessor analog referred to as Vasculotide. Angiopoietins are ligands for the receptor tyrosine kinase Tie2 and Vasculotide has shown dose-dependent binding and phosphorylation of Tie 2 (Gutbier et al., 2017; Dekker et al., 2018). Vasculotide is a Tie2 receptor agonist Angiopoietin-1 (Angpt-1) mimetic that decreases neuroinflammation and BBB leakage and improves neurological functional outcome after stroke in diabetic rats (Venkat et al., 2018, 2021). Angpt-1 is an endothelial growth factor that mediates vascular remodeling (Suri et al., 1996) and promotes pericyte recruitment, remodeling, maturation, and stabilization of blood vessels (Suri et al., 1998; Iurlaro et al., 2003), neurite remodeling (Wang et al., 2015), and prevents plasma leakage in the ischemic brain (Zhang et al., 2002; Metheny-Barlow et al., 2004). Systemic administration of Vasculotide induces long-lasting Tie2 activation, decreases endothelial barrier dysfunction and microvascular leakage, and improves microcirculatory perfusion in rodent models of abdominal sepsis (Kumpers et al., 2011), acute kidney injury (Rübig et al., 2016) and hemorrhagic shock (Trieu et al., 2018). In a rodent model of AD, treatment with Vasculotide accelerates the restoration of the BBB after focused ultrasound-induced permeability (Lynch et al., 2021). In the current study, we investigate the safety and therapeutic effects of AV-001 (clinical candidate version of Vasculotide) in middle-aged rats subject to MMI and evaluate its efficacy in ameliorating cognitive deficits by reducing WM injury.

## Materials and Methods

All procedures were carried out in accordance with the American Council on Animal Care and with the approval of Institutional Animal Care and Use Committee (IACUC, protocol # 1012) of Henry Ford Health System. This manuscript is prepared following ARRIVE guidelines (Kilkenny et al., 2010).

### MMI Model

The method for preparation of cholesterol crystals and the MMI model have been described in detail previously (Rapp et al., 2008a; Venkat et al., 2017; Wang et al., 2017; Yu et al., 2019; Chandran et al., 2020). Briefly, freshly prepared crystals were filtered using a 100 μm cell strainer and filtrate was passed through a 70 μm cell strainer to collect residual crystals of size 70–100 μm. The crystals were counted on a hemocytometer and diluted to yield a final concentration of 800 ± 100 crystals/300 μl saline. Rats were anesthetized with 4% isoflurane in a chamber and then spontaneously respired with 2% isoflurane in 2:1 N_2_O:O_2_ mixture using a facemask connected and regulated with a modified FLUOTEC 3 Vaporizer (Fraser Harlake, NY, USA). Rectal temperature was maintained at 37°C throughout the surgical procedure using a water heating system. A midline neck incision (~1 cm long) was made and the right CCA (common carotid artery), ECA (external carotid artery), and ICA (internal carotid artery) were exposed under an operating microscope. Carefully avoiding the vagus nerve, the CCA and ICA were temporarily clamped using microsurgical clips and a 4–0 silk suture tied loosely at the origin of the ECA and ligated at the distal end of the ECA. A 1 ml syringe connected to a PE-50 tube, with its tip tapered by heating near a flame was gently inserted into the ECA and advanced into the lumen of the ICA. The microsurgical clip repositioned to only block the CCA and freshly prepared cholesterol crystals were slowly injected into the ICA. The tube was gently removed, ECA ligated while CCA and ICA remain patent, microsurgical clips were removed, and the neck incision sutured. Routine post-surgical monitoring, analgesics (Buprenorphine SR, 1 mg/Kg, subcutaneously), and supportive care was provided.

### Experimental Groups, Randomization, and Blinding

Male, 10–12 months old Wistar rats (Charles River Laboratories, MA, USA) were employed. The experimental groups are as follows: (1) MMI (*n* = 20); (2) MMI+ 1 μg/Kg AV-001 (*n* = 20); (3) MMI+ 3 μg/Kg AV-001 (*n* = 15); (4) MMI+ 6 μg/Kg AV-001 (*n* = 8); and (5) Naïve sham control rats (*n* = 20). At 1 day after MMI, rats were randomly assigned to one of the treatment groups. Stock solutions and aliquots of AV-001 were prepared in DPBS and stored in −20°C. AV-001 treatments were initiated at 1 day after MMI and administered (at room temperature) once daily *via* i.p. injection. Rats were sacrificed at 6 weeks after MMI and the number of animals used for various analyses is indicated in Table 1. All end-point measurements including cognitive tests, neurological function evaluation, and immunostaining quantification analysis were performed by investigators blinded to the experimental groups. To overcome practical problems of blinding treatment groups, the investigator performing behavioral testing was not involved in performing surgery or treatment administration. Data analysis was performed by a Biostatistician.

**Table 1:** Rats were sacrificed at 6 weeks after MMI and the number of animals used for various analysis is indicated.

	Sham	MMI	MMI+ 1 μg/Kg AV-001	MMI+ 3 μg/Kg AV-001	MMI+ 6 μg/Kg AV-001
Function testing	20	20	20	15	8
Immunohistochemistry	10	10	10		
Electron microscopy	6	5	5		
Golgi staining	4	5	5		

AV-001 is a synthetic PEGylated peptide conjugate derived from four identical 7-amino-acid peptides (T7) bound to the PEG tetramer. The core T7 peptide used to construct AV-001 was based on its selection from a phage-display approach that examined over 1 billion unique peptide sequences for their ability to bind the extracellular portion of the Tie2 receptor (Tournaire et al., 2004). AV-001 is a clinical candidate version of Vasculotide, an Angpt-1 mimetic peptide. Previous studies using Vasculotide as a therapeutic agent have reported its safety and identified that a dose of 10 μg/Kg reduces skin damage following a single large dose of ionizing radiation in mice (Korpela et al., 2014), 200 ng dose reduces mortality in a murine sepsis model (Kumpers et al., 2011), 500 ng dose is protective in a murine model of severe influenza (Sugiyama et al., 2015), and 3 μg/Kg dose induces neuroprotection in diabetic rats subject to stroke (Venkat et al., 2018, 2021). Vasculotide administered i.p. to healthy mice significantly increases endothelial Tie2 activation up to 72 h after injection and increases plasma levels of Vasculotide at 24 h after injection which declined to basal levels by 96 h after injection (Kumpers et al., 2011; Sugiyama et al., 2015). Based on these studies, we selected and tested three doses of AV-001 (1 μg/Kg, 3 μg/Kg and 6 μg/Kg) and treatment was initiated at 24 h after MMI and administered *via* i.p. injection once daily for a period of 6 weeks.

### Neurological and Cognitive Function Evaluation

To assess neurological deficits, the modified neurological severity score (mNSS) test was performed after MMI on day 1 and weekly once thereafter until sacrifice. The mNSS test is widely used to evaluate neurological function following brain injury in rodents and is a composite of motor, sensory, balance and reflex tests (Chen et al., 2001; Bieber et al., 2019). The absence of a tested reflex or abnormal response is scored as one point. Neurological function is scored between 0 and 18 with 0 indicating no deficits and 18 indicating maximum deficits.

A battery of cognitive tests was performed using ANY-Maze (Stoelting Co., IL, USA) video tracking and analysis system at 4–5 weeks after MMI such that no two tests were performed on the same day. All animals were habituated to the testing environment 1 day prior to testing. To test short-term memory, a novel object recognition test was used (Venkat et al., 2017; Cui et al., 2018). Briefly, animals were allowed to freely explore two identical objects placed equidistant from each other in the center of a testing arena for 5 min. After a retention delay of 4 h, one object was replaced with a novel object and animals freely explored the two objects for 5 min. The time spent exploring each object was recorded. Animals with a total exploration time <10 s were excluded. Exploration was defined as actively sniffing, pawing, or probing with whiskers within 1 cm of an object. The discrimination index was calculated as the ratio of time spent exploring the novel object to total time spent exploring both objects.

To test long-term memory, we employed a novel odor recognition test spanning 3 days during which animals were single housed. Two sets of novel odor beads (N1 and N2) were obtained by placing 1” round wooden beads (BE1090^1^) in the home cage of donor rats for 1 week to allow odor build up. The testing paradigm for the odor test has been described in detail previously (Spinetta et al., 2008; Zhang et al., 2015; Venkat et al., 2017). Briefly, on the first day of testing, four wooden beads were introduced in the home cage of animals to habituate them to presence of beads and to collect familiar odor beads (F). On the second day of testing, animals were familiarized with novel odor N1 by allowing them to freely explore three F beads and one N1 bead for three 1-min trials. The spatial arrangement of N1 and F beads were randomly altered for each trial. Following a retention delay of 24 h i.e., on the third day of testing, the rats were subjected to a 1 min test in which animals explored two familiar odor beads (F), one N1 odor bead and one N2 odor bead introduced into the center of their cage. The trial was video recorded, and the time spent exploring each odor (F, N1, N2) was recorded. The four-choice procedure for assessing relative odor preference greatly increases sensitivity and reliability compared to two-choice procedures (Spinetta et al., 2008). To avoid scent marking, a fresh N1 and N2 bead was used for each trial. The discrimination index was calculated as the ratio of time spent exploring the N2 odor to total time spent exploring all beads. Animals that were inactive and failed to explore any of the beads were excluded.

Spatial learning and memory were evaluated using a Morris water maze test (Zhang et al., 2015; Venkat et al., 2017). The 5 day test consisted of four daily 90-s trials in which the animal searched for a submerged clear platform in a pool of water. The pool was virtually divided into four quadrants and the position of the hidden platform was randomly varied within the target quadrant for each trial. Percentage of time spent in the target quadrant, time taken to reach the hidden platform (escape latency) and swim speed were recorded for each trial and averaged for each day per animal. Greater percentage of time spent in the target platform quadrant and lower escape latency time indicates better learning and memory.

The three-chamber social test (Nadler et al., 2004) was used to evaluate sociability and preference for social novelty. The test is based on the premise that rodents are sociable and prefer to spend more time with another rodent (S1) compared to an empty chamber and given a choice, prefer to interact with a novel stranger (S2) compared to a familiar rodent (S1) i.e., prefer social novelty. S1 and S2 were non-test sham animals of same sex and similar age and weight. The testing apparatus consisted of three proportional plexiglass chambers with openings that allow animals to freely move between the chambers. Prior to testing, animals were placed alone in a holding cage for 30 min since pre-test social deprivation has been reported to increase baseline levels of social behavior and improve sensitivity of the test. During the sociability test, S1 was placed in a wire cup in one chamber while an empty wire cup was placed in the other corner chamber. During the social novelty test, S1 and S2 were each placed in a wire cup in one of the corner chambers. For each test, the rat was introduced in the center chamber and the time spent investigating S1 and empty cup in sociability phase and the time spent investigating S1 and S2 in social novelty phase were recorded. After each test, the apparatus was wiped clean with 3% peroxide hydrochloride and water. Animals that were inactive and did not transition between the chambers were excluded from the analysis (Yang et al., 2011).

### Physiological Measurements and Safety Evaluation

Body weight was recorded weekly. To evaluate the effect of MMI on blood pressure (BP) and heart rate, diastolic arterial pressure, mean arterial pressure, and systolic arterial pressure were measured using the tail-cuff method (CODA 8-Channel High Throughput Non-Invasive Blood Pressure system, KENT Scientific, CT, USA) at baseline, 1 day after MMI, and prior to sacrifice. Liver, kidney, and heart function was assessed by measurement of serum alanine transaminase (ALT) levels using the ALT Activity Assay kit (Sigma-Aldrich, MO, USA, MAK052), serum creatinine levels using the Rat Creatinine Assay kit (Crystal Chem Inc., IL, USA, 80340) and cardiac troponin (cTnI) and Creatine Kinase MB (CK-MB) levels using the i-STAT handheld blood analyzer (Abbott, IL, USA) and cTnI cartridge (CLIAwaived Inc., CA, USA, ABBT-03P90) and CK-MB cartridge (CLIAwaived Inc., CA, USA, ABBT-03P92), respectively, following manufacturer’s instructions.

### Histological and Immunohistochemical Assessment

Rats (*n* = 10/group) were transcardially perfused with 0.9% saline, and brains and hearts were immediately removed, and immersion fixed in 4% paraformaldehyde. Paraffin embedded brain coronal tissue sections were prepared and Bielschowsky silver (axon marker) and Luxol fast blue (myelin marker) staining were used to assess axon and myelin density. Antibodies against APC (oligodendrocyte marker, GenWay Biotech Inc., CA, USA, 1:20), NG2 (oligodendrocyte progenitor cell marker, Millipore Sigma, MA, USA, 1:400), DCX (marker of immature neurons and neurogenesis, Abcam, Cambridge, UK, 1:800), Ki67 (marker of cell proliferation, Lab Vision, CA, USA, 1:200), Synaptophysin (synaptic protein, Abcam, Cambridge, UK, 1:400) and pNFH (phosphorylated neurofilament heavy chain protein, Biolegend, CA, USA, 1:1,000) were also used. Paraffin embedded heart sections (6 μm) were cut and Picrosirius red (PSR) staining was used to evaluate fibrosis by measuring interstitial collagen fraction. Negative controls were processed in a similar fashion, but without adding the primary antibody.

### Quantification Analysis

Although cholesterol crystals are only injected into the right ICA, WM/axonal damage have been consistently observed in both cerebral hemispheres in our study as well as in previous studies using the MMI model (Wang et al., 2012, 2017; Venkat et al., 2017). For each brain section, six fields of view of striatum, cortex, corpus callosum, or subventricular zone (SVZ) were digitized under a 20× objective (Olympus BX40) using a 3-CCD color video camera with an MCID image analysis system (Imaging Research, ON, Canada). For each heart section, 6–8 fields of view were randomly digitized under a 20× objective (Olympus BX40) using a 3-CCD color video camera (Sony DXC-970MD) interfaced with an MCID image analysis system. Using MCID image analysis, the numbers of immunoreactive cells were counted or positive-stained areas were measured (densitometry function) with a density threshold set uniformly above unstained for all groups. For each animal, data were averaged to yield either percentage positive area or number of positive cells/mm^2^.

### Electron Microscopy (EM)

Transmission electron microscopy was employed to analyze the ultrastructure of myelination, as previously described (Venkat et al., 2017; Zhang et al., 2021). The percentage of demyelinated axons, myelin thickness, and G ratio (ratio of axon internal to external diameter) were calculated in the corpus callosum of Sham, MMI, and MMI+1 μg/Kg AV-001 groups (*n* = 5–6/group). Samples from each brain section containing eight fields of view of the corpus callosum were digitized using a JEOL 1400 Flash with a bottom mounted BioSprint camera using AMT image capturing software. Images were analyzed using an MCID image analysis system.

### Golgi Staining

Golgi staining (FD NeuroTechnologies, Inc., MD, USA, Rapid Golgi stain kit, and manufacturer’s protocol was used) was used to evaluate the morphology of neurons, as previously described (Venkat et al., 2017). To evaluate neurite branching, 10 intact neurons were randomly selected from the layer III of the cortex and primary and secondary branching were counted under a 40× objective (Olympus BX40) using a 3-CCD color video camera with an MCID image analysis system. To calculate dendritic spine density, 10 neurons from each brain sample in layer III of the cortex and CA3 region of hippocampus were selected and secondary dendrites measuring at least 10 μm in length were digitized under an oil immersion 100× objective.

### Statistical Analysis

Data are presented as mean ± SEM for illustration. Data were evaluated for normality; ranked data were used for analysis when data were not normally distributed. Analysis of variance (ANOVA) was used for single measurements collected at day 28 including novel object recognition test, novel odor recognition test, social test and immunostaining measurements. Repeated analysis of variance (ANCOVA) was used to test AV-001 dose effects on function recovery for mNSS and Morris water maze tests and body weight considering treatment dose by time interaction. Statistical significance was detected at *p* < 0.05.

## Results

### AV-001 Treatment Is Safe in Middle-Aged Rats Subjected to MMI

**Figure 1:**
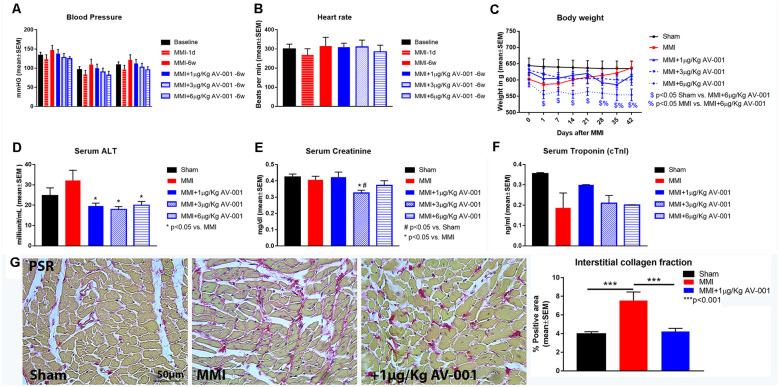
AV-001 treatment is safe in middle-aged rats subject to MMI. **(A,B)** There were no significant differences in blood pressure or heart rate at 1 day or 6 weeks after MMI compared to baseline values. AV-001 treatment (1, 3, or 6 μg/Kg) does not significantly alter blood pressure or heart rate at 6 weeks after MMI compared to baseline values or the MMI control group. **(C)** Treatment of MMI with 1, 3 or 6 μg/Kg AV-001 does not significantly alter body weight at day 42 compared to baseline values before treatment initiation at 1 day after MMI. While 6 μg/Kg AV-001 treated group exhibit significantly lower body weight compared to Sham and MMI control group, the weight difference is evident starting at 1 day after MMI before treatment was initiated and is not significantly different on day 42 compared to its baseline values at day 0 or day 1 after MMI. **(D)** MMI does not alter serum Alanine transaminase (ALT) levels compared to Sham group. AV-001 treatment (1, 3, or 6 μg/Kg) significantly reduces serum ALT levels compared to the control MMI group. **(E)** MMI does not alter serum creatinine levels compared to Sham group. Treatment with 3 μg/Kg AV-001 significantly reduces serum creatinine levels compared to the control MMI group. **(F)** Serum Troponin I (cTnI) levels were within normal range for Sham, MMI, and AV-001 treatment (1, 3, or 6 μg/Kg) groups at 6 weeks after MMI. **(G)** MMI significantly increases fibrosis within the heart as indicated by increased interstitial collagen compared to sham rats. 1 μg/Kg AV-001 treatment significantly reduces collagen in the heart compared to untreated MMI animals.

Early mortality at 1 day after MMI was 5%. There was no mortality or observed adverse effects after 1, 3, or 6 μg/Kg AV-001 treatment in middle-aged rats subjected to MMI. There were no significant differences in BP or HR at 1 day or 6 weeks after MMI compared to baseline values. AV-001 treatment (1, 3, or 6 μg/Kg) does not significantly alter BP or HR at 6 weeks after MMI compared to baseline values or the MMI control group. Treatment of MMI with 1 or 3 μg/Kg AV-001 treatment does not significantly alter body weight compared to Sham or MMI control group. While 6 μg/Kg AV-001 treated group exhibit significantly lower body weight compared to Sham and MMI control group, the weight difference is evident starting at 1 day after MMI before treatment was initiated and is not significantly different on day 42 compared to its baseline values at day 0 or day 1 after MMI. AV-001 treatment (1, 3, or 6 μg/Kg) significantly reduces serum ALT levels, and 3 μg/Kg AV-001 treatment significantly reduces serum creatinine levels compared to the control MMI group, although ALT and creatinine values remain within the normal range (10–40 IU/L and 0.4–0.8 mg/dl, respectively). In addition, cTnI values were within normal range and CK-MB values were 0.0 ng/ml for Sham, MMI and AV-001 treatment (1, 3, or 6 μg/Kg) groups. MMI significantly increases fibrosis within the heart as indicated by increased interstitial collagen compared to sham rats. One microgram per Kg AV-001 treatment significantly reduces collagen in the heart compared to untreated MMI animals. Data are summarized in Figure 1.

### AV-001 Treatment Significantly Improves Cognition and Memory in Middle-Aged Rats Subjected to MMI

**Figure 2:**
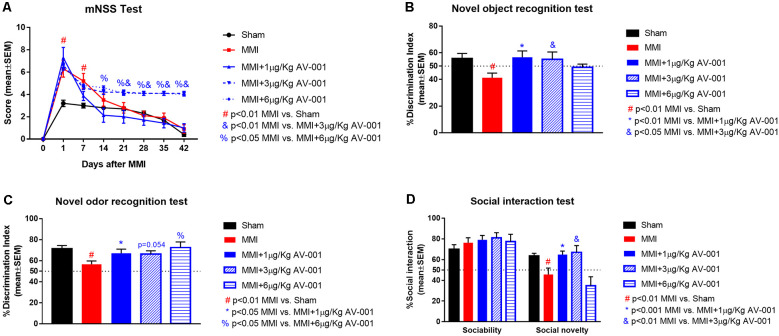
AV-001 treatment significantly improves cognition and memory in middle-aged rats subjected to MMI. **(A)** There were no significant differences in modified neurological severity score (mNSS) after 1 μg/Kg AV-001 treatment compared to the MMI control group. However, 3 or 6 μg/Kg AV-001 treatment group exhibited significantly worse neurological function compared to MMI control group. **(B)** Compared to Sham control rats, middle-aged rats subject to MMI exhibit significant short term memory impairment indicated by lower discrimination index in the novel object recognition test. Rats treated with 1 or 3 μg/Kg AV-001 exhibit significantly improved short-term memory compared to MMI rats. **(C)** Compared to Sham control rats, middle-aged rats subject to MMI exhibit significant long term memory impairment indicated by lower discrimination index in the novel odor recognition test. Rats treated with 1 or 6 μg/Kg AV-001 exhibit significantly improved long-term memory compared to MMI rats. **(D)** Compared to sham control rats, middle-aged rats subject to MMI exhibit significantly reduced preference for social novelty in the social interaction test. Rats treated with 1 or 3 μg/Kg AV-001 exhibit significantly improved preference for social novelty compared to MMI rats.

To identify an optimal dose of AV-001 to treat VaD, middle-aged rats were subjected to MMI and treated once daily with 1, 3, or 6 μg/Kg AV-001 starting at 1 day after MMI. At 1 day after MMI, the mNSS scores were low (5.3 ± 0.22) on a scale of 0–18 (<6 indicating mild to no neurological deficits, 6–12 indicating significant neurological deficits, and >13 severe neurological deficits predictive of poor survival). Such low mNSS scores are unlikely to interfere with cognitive function evaluation. There were no significant differences in mNSS scores after 1 μg/Kg AV-001 treatment compared to the MMI control group, however, 3 or 6 μg/Kg AV-001 treatment groups exhibited significantly worse neurological function compared to the MMI control group on days 14–42 and 21–42 after MMI, respectively (Figure 2A).

A battery of cognitive tests was conducted at 4–5 weeks after MMI (Figures 2, [Fig F3]). We found that MMI in middle-aged rats induces significant cognitive impairment including short-term memory loss, long-term memory deficits, spatial learning and memory deficits, and reduced preference for social novelty when compared to sham control rats. Rats treated with 1 μg/Kg AV-001 exhibit significantly improved short-term memory, long-term memory, spatial learning and memory as well as improved preference for social novelty compared to MMI rats. Treatment with 3 μg/Kg AV-001 improves short-term memory and preference for social novelty but does not improve long-term memory or spatial learning and memory compared to control MMI rats. Treatment with 6 μg/Kg AV-001 improves only long-term memory compared to MMI rats. In addition, 6 μg/Kg AV-001 treated MMI rats exhibit significantly lower swim speeds compared to control MMI rats. A dose is considered effective if there is significant cognitive recovery with no safety concerns in middle-aged rats subjected to MMI, compared to control MMI rats. Therefore, 1 μg/Kg AV-001 was identified as an optimal dose and was used for immunostaining analysis.

**Figure 3 F3:**
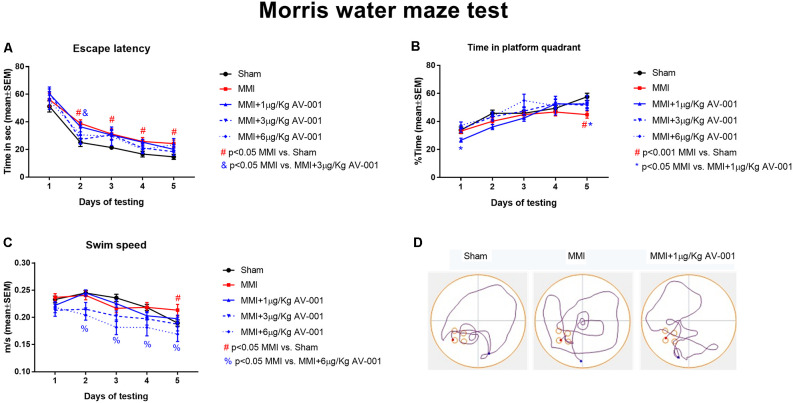
AV-001 treatment significantly improves spatial learning and memory in middle-aged rats subjected to MMI. **(A,B)** In the Morris water maze test, rats subjected to MMI exhibit significant spatial learning and memory impairment indicated higher escape latency and lower percentage of time exploring the platform quadrant compared to Sham control rats. Treatment with 1 μg/Kg AV-001 significantly increases the time spent exploring the platform quadrant on day 5 of testing compared to control MMI rats indicating improved cognition. **(C)** Rats subject to MMI and treated with 6 μg/Kg AV-001 exhibit significantly lower swim speeds compared to control MMI rats. **(D)** Representative track plots of day 5 of testing.

### AV-001 Treatment Significantly Improves WM Integrity and Promotes Axonal/WM Remodeling in Middle-Aged Rats Subjected to MMI

**Figure 4:**
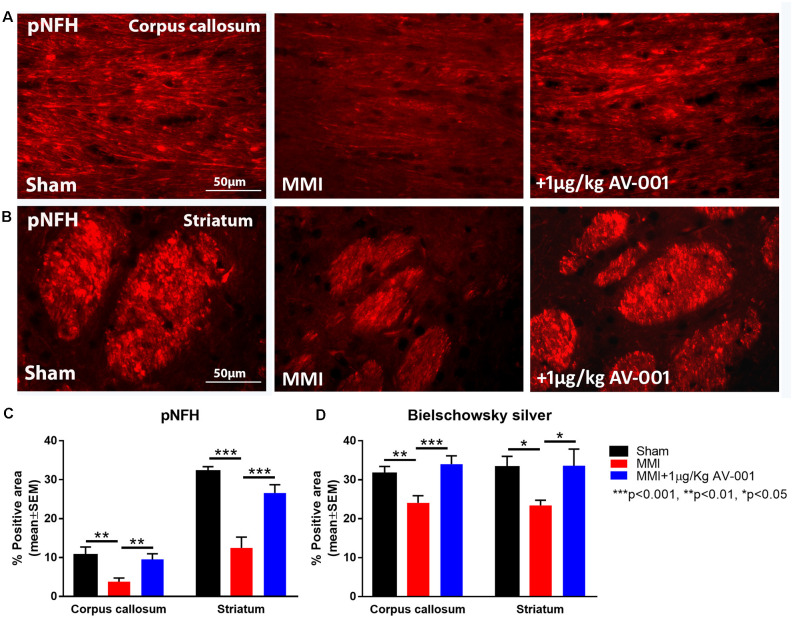
AV-001 treatment significantly improves axonal/white matter remodeling in middle-aged rats subjected to MMI. **(A–C)** Representative images and quantification data of pNFH staining in the Corpus callosum and Striatum. MMI induces significant axon damage in the Corpus callosum and white matter bundles of the Striatum compared to Sham control rats. Treatment with 1 μg/Kg AV-001 significantly increases axon density in the Corpus callosum and Striatum compared to MMI rats. **(D)** Quantification data of Bielschowsky silver staining indicating that MMI decreases axon density compared to Sham control rats, while 1 μg/Kg AV-001 treatment significantly increases axon density in the Corpus callosum and Striatum compared to MMI rats.

**Figure 5:**
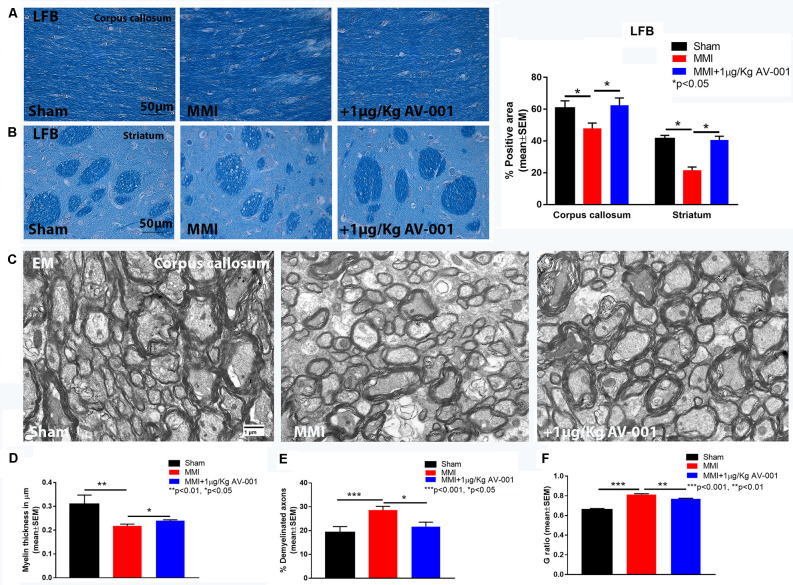
AV-001 treatment significantly improves myelin density in the brain of middle-aged rats subjected to MMI. **(A,B)** Representative images and quantification data of luxol fast blue (LFB) staining in the Corpus callosum and Striatum. MMI significantly reduces myelin density in the Corpus callosum and white matter bundles of the striatum compared to Sham control rats. Treatment with 1 μg/Kg AV-001 significantly increases myelin density in the Corpus callosum and Striatum compared to MMI rats. **(C–F)** Representative images and quantification data of electron microscopy in the corpus callosum. MMI significantly reduces myelin thickness, increases the number of demyelinated axons and increases G-ratio compared to Sham control rats. 1 μg/Kg AV-001 treatment significantly increases myelin thickness, reduces the number of demyelinated axons and reduces G-ratio compared to the MMI control group.

**Figure 6:**
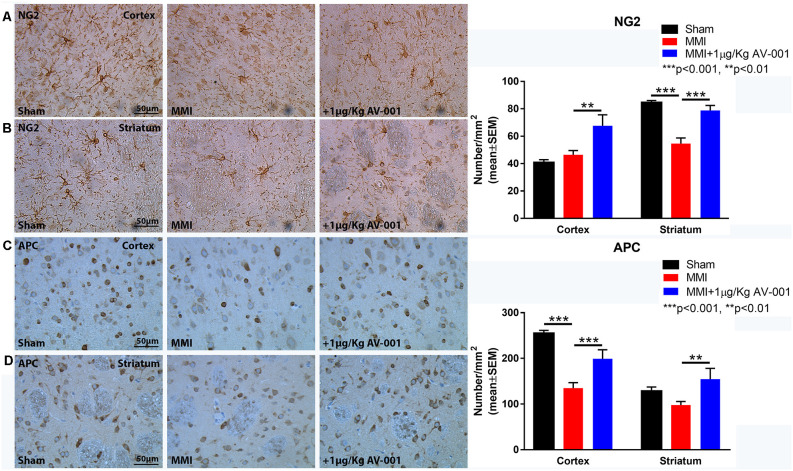
AV-001 treatment significantly increases oligodendrocyte progenitor cells and oligodendrocytes in the brain of middle-aged rats subjected to MMI. **(A,B)** Representative images and quantification data of NG2 immunostaining in the Cortex and Striatum brain regions. MMI significantly reduces the number of oligodendrocyte progenitor cells in the striatum compared to Sham control rats. 1 μg/Kg AV-001 treatment significantly increases the number of oligodendrocyte progenitor cells in the Cortex and Striatum compared to the MMI control group. **(C,D)** Representative images and quantification data of APC immunostaining in the Cortex and Striatum brain regions. MMI significantly reduces the number of oligodendrocytes in the cortex compared to Sham control rats. 1 μg/Kg AV-001 treatment significantly increases the number of oligodendrocytes in the Cortex and Striatum and oligodendrocyte progenitor cells in the striatum compared to the MMI control group.

To test the effect of AV-001 treatment on WM injury, axon and myelin density were evaluated in the corpus callosum and WM bundles in the striatum. Treatment with 1 μg/Kg AV-001 significantly increases axon density (Figure 4) indicated by Bielschowsky silver and pNFH staining in the corpus callosum and striatum as well as increases myelin density (Figures 5A,B) indicated by Luxol fast blue staining in the corpus callosum compared to the MMI control group. We then employed electron microscopy to analyze the ultrastructure of myelination and the percentage of demyelinated axons, myelin thickness and G ratio were calculated in the corpus callosum of Sham, MMI, and MMI+1 μg/Kg AV-001 treated rats. Treatment of middle-aged rats subjected to MMI with 1 μg/Kg AV-001 significantly increases myelin thickness, reduces the number of demyelinated axons and reduces G-ratio compared to the MMI control group (Figures 5C–F). Since oligodendrocyte progenitor cells and oligodendrocytes are required for remyelination after brain injury, we measured the number of oligodendrocyte progenitor cells using NG2 staining and oligodendrocytes using APC staining in the cortex and striatum. Figure 6 shows that 1 μg/Kg AV-001 treatment significantly increases the number of oligodendrocytes in the cortex and striatum and oligodendrocyte progenitor cells in the striatum compared to the MMI control group.

### AV-001 Treatment Significantly Increases Neurogenesis and Synaptogenesis in Middle-Aged Rats Subjected to MMI

**Figure 7:**
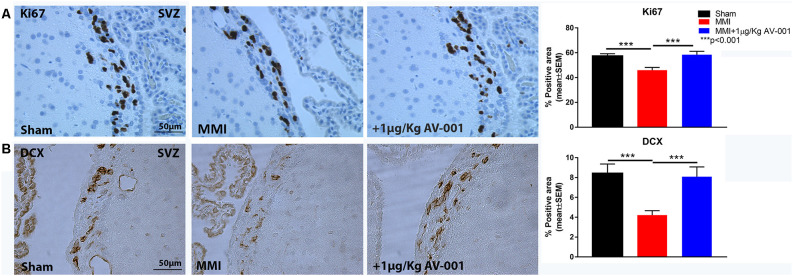
AV-001 treatment significantly increases neurogenesis in middle-aged rats subjected to MMI. **(A)** Representative images and quantification data of Ki67 immunostaining in the sub ventricular zone (SVZ). MMI significantly reduces cell proliferation in the SVZ compared to Sham control rats. Treatment with 1 μg/Kg AV-001 significantly improves cell proliferation in the SVZ compared to control MMI animals. **(B)** Representative images and quantification data of DCX immunostaining in the sub ventricular zone (SVZ) brain region. MMI significantly reduces neurogenesis in the SVZ compared to Sham control rats. Treatment with 1 μg/Kg AV-001 significantly improves neurogenesis in the SVZ compared to control MMI animals.

**Figure 8:**
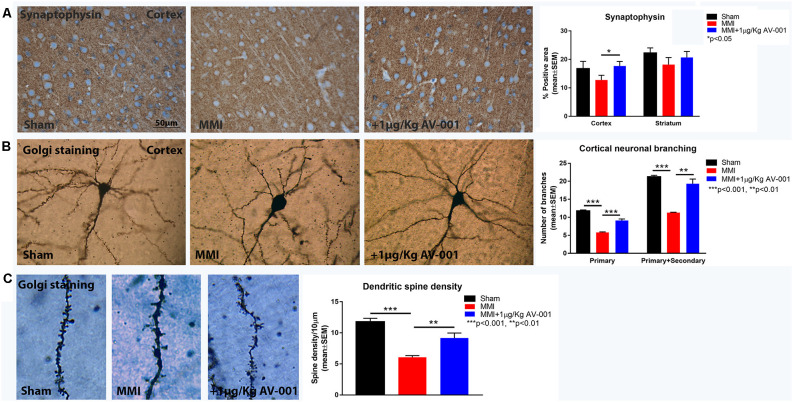
AV-001 treatment significantly increases synaptogenesis in middle-aged rats subjected to MMI. **(A)** Representative images and quantification data of synaptic protein expression in the Cortex and Striatum. Treatment with 1 μg/Kg AV-001 significantly increases Synaptophysin expression in the cortex compared to control MMI animals. **(B,C)** Representative images and quantification data of Golgi staining in the Cortex region of brain. MMI significantly decreases neuronal branching in the layer III of Cortex as well as decreases dendritic spine density compared to Sham control rats. Treatment with 1 μg/Kg AV-001 significantly increases neuronal branching and dendritic spine density in the cortex region compared to control MMI rats.

Neuroplasticity is the ability of the brain to rewire in response to stimulation or injury and is associated with neurogenesis, the ability to create new neurons and synaptogenesis, the formation of connections between neurons. We evaluated neurogenesis in the SVZ using immunostaining analysis of Ki67, a marker of cell proliferation and DCX, a marker of immature neurons (Zhao and van Praag, 2020). Figure 7 shows that MMI significantly reduces neurogenesis and cell proliferation in the SVZ. Treatment with 1 μg/Kg AV-001 significantly improves cell proliferation and neurogenesis in the SVZ compared to control MMI animals. To test the effect of AV-001 treatment on synaptic protein expression, synaptophysin expression was measured in the cortex and striatum of MMI rats. Figure 8A shows that treatment with 1 μg/Kg AV-001 significantly increases Synaptophysin expression in the cortex compared to control MMI animals. To test the effects of AV-001 treatment on neuronal branching and spine density, Golgi silver staining was used. Figures 8B,C shows that MMI significantly decreases neuronal branching in the layer III of the cortex as well as decreases dendritic spine density. Treatment with 1 μg/Kg AV-001 significantly increases neuronal branching and dendritic spine density in the cortex region compared to control MMI rats.

## Discussion

Spontaneous cerebral emboli are common in VaD and AD and contribute to a rapid decline in cognitive function in dementia patients (Purandare et al., 2006, 2012). In VaD, emboli block cerebral microvasculature, reducing blood flow to critical areas for cognition and contributing to the development of microinfarcts in the cortex and striatum (Oliveira-Filho et al., 2018). Prolonged cerebral hypoperfusion is a main contributor to cognitive dysfunction in patients with dementia and other neurodegenerative disorders (Leeuwis et al., 2017; Sweeney et al., 2018). In this study, we demonstrate for the first time that 1 μg/Kg AV-001 treatment of MMI significantly improves short-term memory, long-term memory, social interaction, spatial learning and memory as well as promotes white matter remodeling and neurogenesis in the brain of middle-aged male rats subject to VaD.

Treatment safety and therapeutic efficacy are primary outcome measures in pre-clinical studies investigating novel therapeutics for neurodegenerative diseases. AV-001 is a clinical candidate version of Vasculotide, an Angpt-1 mimetic peptide. In our previous studies, we found that treatment of stroke in mice with type 1 diabetes mellitus (T1DM) with 3 μg/Kg Vasculotide was safe and effective in improving neurological function, decreasing BBB permeability, and reducing neuroinflammation with no evident increase in post-stroke mortality or other adverse effects (Venkat et al., 2018, 2021). Vasculotide treatment has been shown to reduce mortality and improve organ function in rodent models of abdominal sepsis and kidney transplant (Kumpers et al., 2011; Thamm et al., 2016). A single dose of Vasculotide (200 ng/rat, i.v.) treatment did not alter BP, heart rate, pH, and partial pressure of carbon dioxide in a rat hemorrhagic shock and fluid resuscitation model compared with the vehicle treated control group (Trieu et al., 2018). In the current study, we show that AV-001 treatment does not significantly alter body weight, BP, or heart rate at 6 weeks after MMI compared to baseline measurements or the MMI control group. However, rats treated with 3 or 6 μg/Kg AV-001 exhibited significantly worse neurological function compared to control MMI rats. We next tested the levels of ALT, creatinine, cTnI, and CK-MB in the serum as elevated levels are indicative of liver, kidney and cardiac toxicity and dysfunction, respectively. MMI did not significantly alter serum ALT or creatinine levels compared to control rats. AV-001 treatment (1, 3, or 6 μg/Kg) significantly reduces serum ALT levels compared to control MMI rats. In addition, 3 μg/Kg AV-001 treatment significantly reduces serum creatinine levels compared to MMI control rats, which is consistent with previous data on Vasculotide treatment in acute kidney injury (Rübig et al., 2016). In prior work, we reported that mice subjected to a bilateral common carotid artery stenosis (BCAS) model of VaD exhibit significant cardiac dysfunction indicated by reduced left ventricular ejection fraction, increased cardiac fibrosis, and cardiac hypertrophy which may be mediated by increased oxidative stress and inflammation in the heart at 1 month after BCAS (An et al., 2021). Angpt-1 is associated with cardio protection against myocardial and vascular injury, promotes the survival of cardiac myocytes, and reduces interstitial fibrosis in the heart (Nykänen et al., 2003; Dallabrida et al., 2005; Lee et al., 2011). In the present study, we found that MMI significantly increases cardiac fibrosis as indicated by increased interstitial collagen fraction in the heart tissue. Treatment with 1 μg/Kg AV-001 significantly reduces interstitial fibrosis in the heart. Elevated cardiac troponin levels are associated with increased severity of left ventricular diastolic dysfunction, and impairment in ejection fraction and cardiac contractility (Adams et al., 2007; Tanabe et al., 2008). We found that cTnI and CK-MB levels were within normal ranges for Sham, MMI and AV-001 treatment groups. The current data demonstrate that treatment of middle-aged MMI rats with 1 or 3 μg/Kg AV-001 for 6 weeks is safe and does not adversely affect organ function.

Our current data as well as previous work show that MMI in middle aged rats induces significant cognitive impairment including short term memory loss, long term memory loss, reduced preference for social novelty and impaired spatial learning and memory compared to sham control rats (Venkat et al., 2017). Rats treated with 1 μg/Kg AV-001 exhibit significantly improved short-term and long-term memory, increased preference for social novelty, and improved spatial learning and memory compared to MMI rats. While treatment with 3 μg/Kg AV-001 improves short-term memory and preference for social novelty, there were no significant improvements in long-term memory or spatial learning and memory compared to control MMI rats. Treatment with 6 μg/Kg AV-001 improves only long-term memory compared to MMI rats. Thus, 1 μg/Kg AV-001 has a greater therapeutic effect in improving cognitive outcome after MMI compared to 3 or 6 μg/Kg AV-001 treatments. In our previous study, we found that treatment of T1DM-stroke with 3 μg/kg Vasculotide but not 2 μg/kg or 5.5 μg/kg Vasculotide significantly improves neurological function and decreases infarct volume and cell death compared to control T1DM-stroke rats (Venkat et al., 2021). A characteristic bell-shaped dose response has been observed for the Vasculotide which seems to be a common feature associated with angiopoietins and activating Tie2 (Gruber et al., 1995; Cho et al., 2004; Brkovic et al., 2007; Maliba et al., 2008; Van Slyke et al., 2009). It has also been reported that at high doses, Vasculotide can result in a suboptimal activation of Tie2 receptor *in vitro* and decreased biological efficacy *in vivo* (Van Slyke et al., 2009). High concentrations of Vasculotide likely result in inefficient clustering of the Tie2 receptor monomers into high-order complexes (Van Slyke et al., 2009). Thus, 1 μg/Kg AV-001 treatment was identified as an optimal therapeutic dose for VaD in middle-aged rats as it provides significant cognitive recovery without any safety concerns.

Patients with VaD experience alterations in the microvasculature supplying subcortical WM which leads to extensive WM damage including vacuolization, rarefaction, and demyelination (Erkinjuntti et al., 1996; Tanabe et al., 1999; Choi et al., 2020). Periventricular WM damage disrupts neuronal connections to the frontal lobe and is associated with impairment in attention, memory, social cognition, and subjective cognitive function (Sultzer et al., 1995; Kynast et al., 2018). WM injury and its progression is caused in part by damage to oligodendrocytes and impairment of oligodendrocyte progenitor cell survival and function, leading to failure of remyelination (Maki et al., 2013). This progression in WM damage is associated with the progression of cognitive function deficits in VaD (Kynast et al., 2018). Our current data as well as previous data indicate that MMI causes extensive WM damage as evident by reduced axon and myelin density in the corpus callosum and striatum, as well as decreased oligodendrocyte and oligodendrocyte progenitor cell number in the cortex and striatum (Venkat et al., 2017, 2019, 2020; Yu et al., 2019). In the current study, we found that treatment of MMI with 1 μg/Kg AV-001 increases axon and myelin density in the corpus callosum and striatum, as well as reduces the number of demyelinated axons, increases myelin thickness and reduces G-ratio in the corpus callosum compared to control MMI rats. The number of oligodendrocytes and oligodendrocyte progenitor cells were significantly increased following 1 μg/Kg AV-001 treatment in the cortex and striatum compared to control MMI rats.

Ischemia-induced cell death is a common feature which contributes to the progression of VaD. The MMI model induces gradual neuronal loss after microinfarction which contributes to cognitive dysfunction (Wang et al., 2012). Previous studies have demonstrated the benefits of stimulating neurogenesis in ameliorating cognitive deficits in VaD models (Zhang et al., 2010; Kwon et al., 2014; Choi et al., 2016). In the current study, we found that MMI significantly reduces neurogenesis and cell proliferation in the SVZ. Treatment with 1 μg/Kg AV-001 significantly improves neurogenesis in the SVZ compared to control MMI rats. Synaptic plasticity is essential in learning and memory and has been reported to mediate improvements in cognitive function after chronic cerebral hypoperfusion (Yao et al., 2019). Synaptophysin is the most abundant protein in synaptic vesicles and is an important factor in synaptic plasticity (Clare et al., 2010). Previously, we found that MMI significantly reduces Synaptophysin expression, neuronal branching and dendritic spine density in the cortex (Venkat et al., 2019, 2020). Our current data shows that treatment with 1 μg/Kg AV-001 significantly increases Synaptophysin expression, neuronal branching and dendritic spine density in the cortex compared to control MMI group. Improved WM remodeling and the increase in neurogenesis and synaptic plasticity may contribute to cognitive recovery after AV-001 treatment of middle-aged rats subjected to MMI.

### Limitations

We have employed middle-aged rats without prior vascular pathologies to test the safety and efficacy of AV-001 to treat MMI induced VaD. Diabetes mellitus is an established risk factor for VaD and has been reported to increase the risk of dementia by up to 60% (Chatterjee et al., 2016). MMI in diabetic animals induces severe cognitive deficits, WM damage, and microglial activation (Chandran et al., 2020). In our previous study, we have evaluated the therapeutic effects of Vasculotide in an animal model of stroke with diabetic co-morbidity and found that 3 μg/Kg Vasculotide improves neurological function, as well as reduces vascular and WM damage, BBB leakage and neuroinflammation compared to control diabetic stroke rats (Venkat et al., 2018, 2021). Common risk factors of VaD differ between males and female (Akhter et al., 2021). For instance, males haves a higher risk of developing VaD with hyperlipidemia or following cardiovascular disease such as stroke and myocardial infarction, while the risk of VaD from pre-existing conditions such as diabetes, obesity and hypertension is higher for females (Akhter et al., 2021). Future studies are warranted to test the therapeutic effects of AV-001 in male and female middle-aged rats with co-morbidities as well as to investigate the molecular mechanisms of AV-001 in VaD.

## Conclusions

In the current study, we found that AV-001 treatment of MMI in male, middle-aged rats is safe, and identified 1 μg/Kg AV-001 as an optimal therapeutic dose to improve cognitive function. Treatment of MMI with 1 μg/Kg AV-001 improves axon density, remyelination, and neuroplasticity in the brain of middle-aged rats subject to MMI.

## Data Availability Statement

The original contributions presented in the study are included in the article, further inquiries can be directed to the corresponding author.

## Ethics Statement

The animal study was reviewed and approved by Institutional Animal Care and Use Committee of Henry Ford Health System.

## Author Contributions

LC, BP, AZ, JL-W, HG, EF, AM, ML, and PV: formal analysis and investigation. PV, LC, and MC: manuscript preparation. PV and MC: conceptualization and supervision. PV: funding acquisition. All authors contributed to the article and approved the submitted version.

## Conflict of Interest

The authors declare that the research was conducted in the absence of any commercial or financial relationships that could be construed as a potential conflict of interest.

## Publisher’s Note

All claims expressed in this article are solely those of the authors and do not necessarily represent those of their affiliated organizations, or those of the publisher, the editors and the reviewers. Any product that may be evaluated in this article, or claim that may be made by its manufacturer, is not guaranteed or endorsed by the publisher.

## Footnotes

^1^ www.craftparts.com

## References

[B1] Adams V. Linke A. Wisloff U. Döring C. Erbs S. Kränkel N.. (2007). Myocardial expression of Murf-1 and MAFbx after induction of chronic heart failure: effect on myocardial contractility. Cardiovasc. Res. 73, 120–129. 10.1016/j.cardiores.2006.10.026 17145048

[B2] Akhter F. Persaud A. Zaokari Y. Zhao Z. Zhu D. (2021). Vascular dementia and underlying sex differences. Front. Aging Neurosci. 13:720715. 10.3389/fnagi.2021.720715 34566624 PMC8457333

[B3] An L. Chopp M. Zacharek A. Shen Y. Chen Z. Qian Y.. (2021). Cardiac dysfunction in a mouse vascular dementia model of bilateral common carotid artery stenosis. Front. Cardiovasc. Med. 8:681572. 10.3389/fcvm.2021.681572 34179145 PMC8225957

[B4] Bieber M. Gronewold J. Scharf A. C. Schuhmann M. K. Langhauser F. Hopp S.. (2019). Validity and reliability of neurological scores in mice exposed to middle cerebral artery occlusion. Stroke 50, 2875–2882. 10.1161/STROKEAHA.119.026652 31412755

[B5] Brkovic A. Pelletier M. Girard D. Sirois M. G. (2007). Angiopoietin chemotactic activities on neutrophils are regulated by PI-3K activation. J. Leukoc. Biol. 81, 1093–1101. 10.1189/jlb.0906580 17215522

[B6] Chandran R. Li W. Ahmed H. A. Dong G. Ward R. A. He L.. (2020). Diabetic rats are more susceptible to cognitive decline in a model of microemboli-mediated vascular contributions to cognitive impairment and dementia. Brain Res. 1749:147132. 10.1016/j.brainres.2020.147132 33002484 PMC7606832

[B7] Chatterjee S. Peters S. A. Woodward M. Mejia Arango S. Batty G. D. Beckett N.. (2016). Type 2 diabetes as a risk factor for dementia in women compared with men: a pooled analysis of 2.3 million people comprising more than 100,000 cases of dementia. Diabetes Care 39, 300–307. 10.2337/dc15-1588 26681727 PMC4722942

[B8] Chen J. Sanberg P. R. Li Y. Wang L. Lu M. Willing A. E.. (2001). Intravenous administration of human umbilical cord blood reduces behavioral deficits after stroke in rats. Stroke 32, 2682–2688. 10.1161/hs1101.098367 11692034

[B9] Cho C. H. Kammerer R. A. Lee H. J. Steinmetz M. O. Ryu Y. S. Lee S. H.. (2004). COMP-Ang1: a designed angiopoietin-1 variant with nonleaky angiogenic activity. Proc. Natl. Acad. Sci. U S A 101, 5547–5552. 10.1073/pnas.0307574101 15060279 PMC397420

[B10] Choi D. H. Lee K. H. Lee J. (2016). Effect of exercise-induced neurogenesis on cognitive function deficit in a rat model of vascular dementia. Mol. Med. Rep. 13, 2981–2990. 10.3892/mmr.2016.4891 26934837 PMC4805106

[B11] Choi H.-I. Ryu C.-W. Kim S. Rhee H.Y. Jahng G.-H. (2020). Changes in microvascular morphology in subcortical vascular dementia: a study of vessel size magnetic resonance imaging. Front. Neurol. 11:545450. 10.3389/fneur.2020.545450 33192974 PMC7658467

[B12] Clare R. King V. G. Wirenfeldt M. Vinters H. V. (2010). Synapse loss in dementias. J. Neurosci. Res. 88, 2083–2090. 10.1002/jnr.22392 20533377 PMC3068914

[B13] Cui G. H. Wu J. Mou F. F. Xie W. H. Wang F. B. Wang Q. L.. (2018). Exosomes derived from hypoxia-preconditioned mesenchymal stromal cells ameliorate cognitive decline by rescuing synaptic dysfunction and regulating inflammatory responses in APP/PS1 mice. FASEB J. 32, 654–668. 10.1096/fj.201700600R 28970251

[B14] Dallabrida S. M. Ismail N. Oberle J. R. Himes B. E. Rupnick M. A. (2005). Angiopoietin-1 promotes cardiac and skeletal myocyte survival through integrins. Circ. Res. 96, e8–24. 10.1161/01.RES.0000158285.57191.60 15692086

[B15] Dekker N. A. M. van Meurs M. van Leeuwen A. L. I. Hofland H. M. van Slyke P. Vonk A. B. A.. (2018). Vasculotide, an angiopoietin-1 mimetic, reduces pulmonary vascular leakage and preserves microcirculatory perfusion during cardiopulmonary bypass in rats. Br. J. Anaesth. 121, 1041–1051. 10.1016/j.bja.2018.05.049 30336848

[B16] Erkinjuntti T. Benavente O. Eliasziw M. Munoz D. G. Sulkava R. Haltia M.. (1996). Diffuse vacuolization (spongiosis) and arteriolosclerosis in the frontal white matter occurs in vascular dementia. Arch. Neurol. 53, 325–332. 10.1001/archneur.1996.00550040053014 8929154

[B17] Goldberg I. Auriel E. Russell D. Korczyn A. D. (2012). Microembolism, silent brain infarcts and dementia. J. Neurol. Sci. 322, 250–253. 10.1016/j.jns.2012.02.021 22429666

[B18] Gorelick P. B. Scuteri A. Black S. E. Decarli C. Greenberg S. M. Iadecola C.. (2011). Vascular contributions to cognitive impairment and dementia: a statement for healthcare professionals from the american heart association/american stroke association. Stroke 42, 2672–2713. 10.1161/STR.0b013e3182299496 21778438 PMC3778669

[B19] Gruber B. L. Marchese M. J. Kew R. (1995). Angiogenic factors stimulate mast-cell migration. Blood 86, 2488–2493. 10.1182/blood.V86.7.2488.2488 7545457

[B20] Gutbier B. Jiang X. Dietert K. Ehrler C. Lienau J. Van Slyke P.. (2017). Vasculotide reduces pulmonary hyperpermeability in experimental pneumococcal pneumonia. Crit. Care 21:274. 10.1186/s13054-017-1851-6 29132435 PMC5683375

[B22] Iurlaro M. Scatena M. Zhu W. H. Fogel E. Wieting S. L. Nicosia R. F. (2003). Rat aorta-derived mural precursor cells express the Tie2 receptor and respond directly to stimulation by angiopoietins. J. Cell Sci. 116, 3635–3643. 10.1242/jcs.00629 12876214

[B23] Kilkenny C. Browne W. Cuthill I. C. Emerson M. Altman D. G. (2010). Animal research: reporting *in vivo* experiments: the ARRIVE guidelines. Br. J. Pharmacol. 160, 1577–1579. 10.1111/j.1476-5381.2010.00872.x 20649561 PMC2936830

[B24] Korpela E. Yohan D. Chin L. C. L. Kim A. Huang X. Sade S.. (2014). Vasculotide, an Angiopoietin-1 mimetic, reduces acute skin ionizing radiation damage in a preclinical mouse model. BMC Cancer 14:614. 10.1186/1471-2407-14-614 25159192 PMC4159535

[B25] Kumpers P. Gueler F. David S. Slyke P. V. Dumont D. J. Park J. K.. (2011). The synthetic tie2 agonist peptide vasculotide protects against vascular leakage and reduces mortality in murine abdominal sepsis. Crit. Care 15:R261. 10.1186/cc10523 22040774 PMC3334812

[B26] Kwon K. J. Kim M. K. Lee E. J. Kim J. N. Choi B. R. Kim S. Y.. (2014). Effects of donepezil, an acetylcholinesterase inhibitor, on neurogenesis in a rat model of vascular dementia. J. Neurol. Sci. 347, 66–77. 10.1016/j.jns.2014.09.021 25266713

[B27] Kynast J. Lampe L. Luck T. Frisch S. Arelin K. Hoffmann K. T.. (2018). White matter hyperintensities associated with small vessel disease impair social cognition beside attention and memory. J. Cereb. Blood Flow Metab. 38, 996–1009. 10.1177/0271678X17719380 28685621 PMC5999004

[B28] Lee S. W. Won J. Y. Lee H. Y. Lee H. J. Youn S. W. Lee J. Y.. (2011). Angiopoietin-1 protects heart against ischemia/reperfusion injury through VE-cadherin dephosphorylation and myocardiac integrin-β1/ERK/caspase-9 phosphorylation cascade. Mol. Med. 17, 1095–1106. 10.2119/molmed.2011.00106 21738954 PMC3188883

[B29] Leeuwis A. E. Benedictus M. R. Kuijer J. P. A. Binnewijzend M. A. A. Hooghiemstra A. M. Verfaillie S. C. J.. (2017). Lower cerebral blood flow is associated with impairment in multiple cognitive domains in Alzheimer’s disease. Alzheimers Dement. 13, 531–540. 10.1016/j.jalz.2016.08.013 27693109

[B30] Lynch M. Heinen S. Markham-Coultes K. O’Reilly M. Van Slyke P. Dumont D. J.. (2021). Vasculotide restores the blood-brain barrier after focused ultrasound-induced permeability in a mouse model of Alzheimer’s disease. Int. J. Med. Sci. 18, 482–493. 10.7150/ijms.36775 33390817 PMC7757142

[B31] Maki T. Liang A. C. Miyamoto N. Lo E. H. Arai K. (2013). Mechanisms of oligodendrocyte regeneration from ventricular-subventricular zone-derived progenitor cells in white matter diseases. Front. Cell Neurosci. 7:275. 10.3389/fncel.2013.00275 24421755 PMC3872787

[B32] Maliba R. Brkovic A. Neagoe P.-É. Villeneuve L. R. Sirois M. G. (2008). Angiopoietin-mediated endothelial P-selectin translocation: cell signaling mechanisms. J. Leukoc. Biol. 83, 352–360. 10.1189/jlb.0107056 17984290

[B33] Metheny-Barlow L. J. Tian S. Hayes A. J. Li L. Y. (2004). Direct chemotactic action of angiopoietin-1 on mesenchymal cells in the presence of VEGF. Microvasc. Res. 68, 221–230. 10.1016/j.mvr.2004.08.005 15501241

[B34] Nadler J. J. Moy S. S. Dold G. Trang D. Simmons N. Perez A.. (2004). Automated apparatus for quantitation of social approach behaviors in mice. Genes Brain Behav. 3, 303–314. 10.1111/j.1601-183X.2004.00071.x 15344923

[B35] Nykänen A. I. Krebs R. Saaristo A. Turunen P. Alitalo K. Ylä-Herttuala S.. (2003). Angiopoietin-1 protects against the development of cardiac allograft arteriosclerosis. Circulation 107, 1308–1314. 10.3390/jpm12020251 12628953

[B36] Oliveira-Filho J. Ay H. Shoamanesh A. Park K. Y. Avery R. Sorgun M.. (2018). Incidence and etiology of microinfarcts in patients with ischemic stroke. J. Neuroimaging 28, 406–411. 10.1111/jon.12512 29607570

[B37] Purandare N. Burns A. Daly K. J. Hardicre J. Morris J. Macfarlane G.. (2006). Cerebral emboli as a potential cause of Alzheimer’s disease and vascular dementia: case-control study. BMJ 332, 1119–1124. 10.1136/bmj.38814.696493.AE 16648133 PMC1459546

[B38] Purandare N. Burns A. Morris J. Perry E. P. Wren J. McCollum C. (2012). Association of cerebral emboli with accelerated cognitive deterioration in Alzheimer’s disease and vascular dementia. Am. J. Psychiatry 169, 300–308. 10.1176/appi.ajp.2011.11010009 22193532

[B39] Rapp J. H. Hollenbeck K. Pan X. M. (2008a). An experimental model of lacunar infarction: embolization of microthrombi. J. Vasc. Surg. 48, 196–200. 10.1016/j.jvs.2008.01.038 18486421

[B40] Rapp J. H. Pan X. M. Neumann M. Hong M. Hollenbeck K. Liu J. (2008b). Microemboli composed of cholesterol crystals disrupt the blood-brain barrier and reduce cognition. Stroke 39, 2354–2361. 10.1161/STROKEAHA.107.496737 18566307

[B41] Rübig E. Stypmann J. Van Slyke P. Dumont D. J. Spieker T. Buscher K.. (2016). The synthetic tie2 agonist peptide vasculotide protects renal vascular barrier function in experimental acute kidney injury. Sci. Rep. 6:22111. 10.1038/srep22111 26911791 PMC4766468

[B42] Spinetta M. J. Woodlee M. T. Feinberg L. M. Stroud C. Schallert K. Cormack L. K.. (2008). Alcohol-induced retrograde memory impairment in rats: prevention by caffeine. Psychopharmacology (Berl) 201, 361–371. 10.1007/s00213-008-1294-5 18758756

[B43] Sugiyama M. G. Armstrong S. M. Wang C. Hwang D. Leong-Poi H. Advani A.. (2015). The Tie2-agonist Vasculotide rescues mice from influenza virus infection. Sci. Rep. 5:11030. 10.1038/srep11030 26046800 PMC4457136

[B45] Sultzer D. L. Mahler M. E. Cummings J. L. Van Gorp W. G. Hinkin C. H. Brown C. (1995). Cortical abnormalities associated with subcortical lesions in vascular dementia. Clinical and position emission tomographic findings. Arch. Neurol. 52, 773–780. 10.1001/archneur.1995.00540320049012 7639629

[B46] Summers P. M. Hartmann D. A. Hui E. S. Nie X. Deardorff R. L. McKinnon E. T.. (2017). Functional deficits induced by cortical microinfarcts. J. Cereb. Blood Flow Metab. 37, 3599–3614. 10.1177/0271678X16685573 28090802 PMC5669342

[B47] Suri C. Jones P. F. Patan S. Bartunkova S. Maisonpierre P. C. Davis S.. (1996). Requisite role of angiopoietin-1, a ligand for the TIE2 receptor, during embryonic angiogenesis. Cell 87, 1171–1180. 10.1016/s0092-8674(00)81813-9 8980224

[B48] Suri C. McClain J. Thurston G. McDonald D. M. Zhou H. Oldmixon E. H.. (1998). Increased vascularization in mice overexpressing angiopoietin-1. Science 282, 468–471. 10.1126/science.282.5388.468 9774272

[B49] Sweeney M. D. Kisler K. Montagne A. Toga A. W. Zlokovic B. V. (2018). The role of brain vasculature in neurodegenerative disorders. Nat. Neurosci. 21, 1318–1331. 10.1038/s41593-018-0234-x 30250261 PMC6198802

[B51] Tanabe M. Crago E. A. Suffoletto M. S. Hravnak M. Frangiskakis J. M. Kassam A. B.. (2008). Relation of elevation in cardiac troponin I to clinical severity, cardiac dysfunction and pulmonary congestion in patients with subarachnoid hemorrhage. Am. J. Cardiol. 102, 1545–1550. 10.1016/j.amjcard.2008.07.053 19026312 PMC3666562

[B50] Tanabe J. L. Ezekiel F. Jagust W. J. Reed B. R. Norman D. Schuff N.. (1999). Magnetization transfer ratio of white matter hyperintensities in subcortical ischemic vascular dementia. Am. J. Neuroradiol. 20, 839–844. 10369354 PMC1892905

[B52] Thamm K. Njau F. Van Slyke P. Dumont D. J. Park J. K. Haller H.. (2016). Pharmacological Tie2 activation in kidney transplantation. World J. Transplant. 6, 573–582. 10.5500/wjt.v6.i3.573 27683636 PMC5036127

[B53] Tournaire R. Simon M. P. le Noble F. Eichmann A. England P. Pouysségur J. (2004). A short synthetic peptide inhibits signal transduction, migration and angiogenesis mediated by Tie2 receptor. EMBO Rep. 5, 262–267. 10.1038/sj.embor.7400100 14978510 PMC1299011

[B54] Trieu M. van Meurs M. van Leeuwen A. L. I. Van Slyke P. Hoang V. Geeraedts J. L. M. G.. (2018). Vasculotide, an angiopoietin-1 mimetic, restores microcirculatory perfusion and Microvascular leakage and decreases fluid resuscitation requirements in hemorrhagic shock. Anesthesiology 128, 361–374. 10.1097/ALN.0000000000001907 28968277

[B55] Van Slyke P. Alami J. Martin D. Kuliszewski M. Leong-Poi H. Sefton M. V.. (2009). Acceleration of diabetic wound healing by an angiopoietin peptide mimetic. Tissue Eng. Part A 15, 1269–1280. 10.1089/ten.tea.2007.0400 18939935

[B56] Venkat P. Chopp M. Chen J. (2015). Models and mechanisms of vascular dementia. Exp. Neurol. 272, 97–108. 10.1016/j.expneurol.2015.05.00 25987538 PMC4631710

[B57] Venkat P. Chopp M. Zacharek A. Cui C. Landschoot-Ward J. Qian Y.. (2019). Sildenafil treatment of vascular dementia in aged rats. Neurochem. Int. 127, 103–112. 10.1016/j.neuint.2018.12.015 30592970

[B58] Venkat P. Chopp M. Zacharek A. Cui C. Zhang L. Li Q.. (2017). White matter damage and glymphatic dysfunction in a model of vascular dementia in rats with no prior vascular pathologies. Neurobiol. Aging 50, 96–106. 10.1016/j.neurobiolaging.2016.11.002 27940353 PMC5209254

[B59] Venkat P. Culmone L. Chopp M. Landschoot-Ward J. Wang F. Zacharek A.. (2020). HUCBC treatment improves cognitive outcome in rats with vascular dementia. Front. Aging Neurosci. 12:258. 10.3389/fnagi.2020.00258 32973489 PMC7461871

[B60] Venkat P. Ning R. Zacharek A. Culmone L. Liang L. Landschoot-Ward J.. (2021). Treatment with an Angiopoietin-1 mimetic peptide promotes neurological recovery after stroke in diabetic rats. CNS Neurosci. Ther. 27, 48–59. 10.1111/cns.13541 33346402 PMC7804913

[B61] Venkat P. Yan T. Chopp M. Zacharek A. Ning R. Van Slyke P.. (2018). Angiopoietin-1 mimetic peptide promotes neuroprotection after stroke in type 1 diabetic rats. Cell Transplant. 27, 1744–1752. 10.1177/0963689718791568 30124060 PMC6300775

[B64] Wang L. Chopp M. Jia L. Lu X. Szalad A. Zhang Y.. (2015). Therapeutic benefit of extended thymosin β4 treatment is independent of blood glucose level in mice with diabetic peripheral neuropathy. J. Diabetes Res. 2015:173656. 10.1155/2015/173656 25945352 PMC4405294

[B65] Wang M. Ding F. Deng S. Guo X. Wang W. Iliff J. J.. (2017). Focal solute trapping and global glymphatic pathway impairment in a murine model of multiple microinfarcts. J. Neurosci. 37, 2870–2877. 10.1523/JNEUROSCI.2112-16.2017 28188218 PMC5354332

[B67] Wang M. Iliff J. J. Liao Y. Chen M. J. Shinseki M. S. Venkataraman A.. (2012). Cognitive deficits and delayed neuronal loss in a mouse model of multiple microinfarcts. J. Neurosci. 32, 17948–17960. 10.1523/JNEUROSCI.1860-12.2012 23238711 PMC3541041

[B68] Yang M. Silverman J. L. Crawley J. N. (2011). Automated three-chambered social approach task for mice. Curr. Protoc. Neurosci. 56, 8.26.1–8.26.16. 10.1002/0471142301.ns0826s56 21732314 PMC4904775

[B69] Yao Z. H. Yao X. L. Zhang S. F. Hu J. C. Zhang Y. (2019). Tripchlorolide may improve spatial cognition dysfunction and synaptic plasticity after chronic cerebral hypoperfusion. Neural Plast. 2019:2158285. 10.1155/2019/2158285 30923551 PMC6409048

[B70] Yu P. Venkat P. Chopp M. Zacharek A. Shen Y. Ning R.. (2019). Role of microRNA-126 in vascular cognitive impairment in mice. J. Cereb. Blood Flow Metab. 39, 2497–2511. 10.1177/0271678X18800593 30215264 PMC6893975

[B72] Zhang Y. Chopp M. Meng Y. Zhang Z. G. Doppler E. Winter S.. (2015). Cerebrolysin improves cognitive performance in rats after mild traumatic brain injury. J. Neurosurg. 122, 843–855. 10.3171/2014.11.JNS14271 25614944

[B73] Zhang Y. Li C. Qin Y. Cepparulo P. Millman M. Chopp M.. (2021). Small extracellular vesicles ameliorate peripheral neuropathy and enhance chemotherapy of oxaliplatin on ovarian cancer. J. Extracell. Vesicles 10:e12073. 10.1002/jev2.12073 33728031 PMC7931803

[B71] Zhang T. Yang Q. W. Wang S. N. Wang J. Z. Wang Q. Wang Y.. (2010). Hyperbaric oxygen therapy improves neurogenesis and brain blood supply in piriform cortex in rats with vascular dementia. Brain Inj. 24, 1350–1357. 10.3109/02699052.2010.504525 20715898

[B74] Zhang Z. G. Zhang L. Croll S. D. Chopp M. (2002). Angiopoietin-1 reduces cerebral blood vessel leakage and ischemic lesion volume after focal cerebral embolic ischemia in mice. Neuroscience 113, 683–687. 10.1016/s0306-4522(02)00175-6 12150788

[B76] Zhao Y. Gan Y. Xu G. Yin G. Liu D. (2020). MSCs-derived exosomes attenuate acute brain injury and inhibit microglial inflammation by reversing CysLT2R-ERK1/2 mediated microglia M1 polarization. Neurochem. Res. 45, 1180–1190. 10.1007/s11064-020-02998-0 32112178

[B75] Zhao X. van Praag H. (2020). Steps towards standardized quantification of adult neurogenesis. Nat. Commun. 11:4275. 10.1038/s41467-020-18046-y 32848155 PMC7450090

[B77] Zhu L. Hoffmann A. Wintermark M. Pan X. Tu R. Rapp J. H. (2012). Do microemboli reach the brain penetrating arteries? J. Surg. Res. 176, 679–683. 10.1016/j.jss.2011.09.059 22261594

